# Women’s experiences of using vaginal trainers (dilators) to treat vaginal penetration difficulties diagnosed as vaginismus: a qualitative interview study

**DOI:** 10.1186/s12905-015-0201-6

**Published:** 2015-06-20

**Authors:** Kat Macey, Angela Gregory, David Nunns, Roshan das Nair

**Affiliations:** Clinical Psychology, University of Nottingham, Nottingham, UK; Chandos Clinic, Nottingham University Hospitals NHS Trust, Nottingham, UK; Division of Obstetrics & Gynaecology, Nottingham University Hospitals NHS Trust, Nottingham, UK; Dept. of Clinical Psychology & Neuropsychology, Nottingham University Hospitals NHS Trust, Nottingham, UK; Clinical Psychology, Division of Psychiatry & Applied Psychology, University of Nottingham, YANG Fujia Building, B Floor, Jubilee Campus, Wollaton Road, Nottingham, NG8 1BB UK

**Keywords:** Vaginismus, Vaginal trainers, Dilators

## Abstract

**Background:**

Recent research has highlighted controversies in the conceptualisation, diagnosis and treatment of vaginismus. Vaginal trainers are currently the most widely used treatment. Critiques have highlighted concerns that the evidence-base of its effectiveness is limited, with controlled trials reporting disappointing results, and its prescription promotes ‘performance-based’ sexuality which may be detrimental. Despite this, little has been done to seek women’s views about their treatment. This study set out to explore women’s experiences of vaginismus treatment with vaginal trainers, and to use their voices to propose guidelines for improving treatment.

**Methods:**

13 women who had used vaginal trainers for vaginal penetration difficulties diagnosed as vaginismus were recruited through a specialist clinic, university campuses, and online forums. The women took part in semi-structured individual interviews (face-to-face/telephone/Skype), which were audio-recorded, transcribed verbatim and analysed using Thematic Analysis.

**Results:**

Four superordinate themes were elicited and used to draft ‘better treatment’ guidelines. Themes were: (1) Lack of knowledge, (2) Invalidation of suffering by professionals, (3) Difficult journey, and (4) Making the journey easier. This paper describes themes (3) and (4). Difficult Journey describes the long and arduous ‘Journey into treatment’, including difficulties *asking for help,* undergoing *physical investigations* and *negotiating ‘the system’* of medical referrals. It also describes the sometimes demoralising process of ‘being in treatment’, which includes *emotional* and *practical demands of treatment*. Making the journey easier highlights *the importance of* and *limits to* ‘partner support’. ‘Professional support’ comprises *personal qualities of professionals/therapeutic relationship*, the value of *specialist skills and knowledge* and the need for *facilitating couple communication about vaginismus*. ‘Peer support/helping each other’ describes the importance of supportive vaginimus *networks* and sharing *tips* with other women.

**Conclusions:**

Accessing effective treatment for vaginal penetration difficulties is difficult. The practical and emotional demands of using vaginal trainers may be underestimated by professionals, resulting in inadequate provision of support and information in practice. At times vaginal trainers may be prescribed to women who are unlikely to benefit from this treatment in isolation. Core communication skills like non-judgemental listening are important for supporting women through treatment. However professionals also need greater specialist knowledge, which in turn requires more detailed research. New ways to disseminate specialist knowledge and suggestions for further research are discussed.

**Electronic supplementary material:**

The online version of this article (doi:10.1186/s12905-015-0201-6) contains supplementary material, which is available to authorized users.

## Background

Vaginismus is often described as a muscular spasm of the outer third of the vagina, causing sex to be painful or impossible. However, investigations into the nature of the proposed spasm have shown that it is not reliably elicited during internal examination [1], and the hypertonicity impeding penetration may not be spasmodic in nature [2]; Difficulty distinguishing between vaginismus and dyspareunia has led to the argument they are not distinct conditions [3, 4]. In response to this The American Psychiatric Association have replaced the diagnoses of vaginismus and dyspareunia with a single diagnosis ‘genito-pelvic pain/penetration disorder’ in their most recent Diagnostic and Statistical Manual (DSM-5) [5]. It is not clear whether this move will be followed in the forthcoming edition of the World Health Organisation’s International Classification of Diseases (ICD-11).

While adoption of GPPPD has been welcomed generally by members of the research community, there are concerns that research is hindered by the failure to differentiate between women who can tolerate attempts at intercourse despite fear and discomfort and between women for whom disgust, fear and avoidance have prevented them from ever having intercourse, and a specifier of ‘never having been able to have intercourse’ has been suggested [6]. There is a lack of clarity in the literature concerning the specific symptoms of women treated. There is clearly a difference between lifelong penetration difficulties related to fear and avoidance, which when overcome result in a woman learning that sex is not painful [7] and the development of difficulties with penetration associated with finding attempted penetration painful, which is more easily accounted for in the new GPPPD diagnosis. Both groups of women could be diagnosed with vaginismus under the ICD-10 criteria and previous DSM-IV-TR criteria.

Using Vaginal Trainers (VTs), also known as dilators, of gradually increasing size to treat vaginismus is widely regarded as one of the ‘classic success stories’ of sex therapy despite a lack of systematic research [8]. Reviews over the last 15 years have indicated an ongoing need for more and better research, however VTs remain the most widely recommended treatment for vaginismus [9–13]. VTs have been used within several vaginismus treatment paradigms, from Sims surgical approach [9], to Masters and Johnson’s couple sex therapy [14], and more recently, individual cognitive behavioural therapy (CBT) approaches [15]. The Masters and Johnson approach has been conceptualised as behavioural exposure treatment, however systemic ideas were also integral to their therapy (the couple’s relationship was considered to be the patient, rather than each individual). Modern CBT approaches often cite Masters and Johnson, but focus on individuals and their cognitions rather than couple interactions. Such differences in the philosophical underpinnings of treatment have rarely been made explicit. Understanding the implications of these differences for treatment is further complicated by lack of clarity and detail in treatment reporting. The role and purported mechanism of vaginal trainers in the treatment of vaginismus has ranged from stretching of the muscles after surgical division, reconditioning (retraining) muscles within an enhanced relationship, to educating a woman that beliefs underlying her fear of penetration are incorrect.

There are few high quality studies investigating the effectiveness of VT treatment for vaginismus, and results have been mixed. A recent randomised controlled trial (RCT) of CBT including behavioural exposure with VTs revealed smaller treatment effects than expected [15], perhaps because partners were not involved. However, a study investigating therapist aided exposure for women with lifelong vaginismus, found that 31 out of 35 women treated were able to have intercourse at follow-up compared to only 4 in the control group [16]. Concerns have been expressed that evaluating treatment in terms of ‘successful penetration’ elevates performance at the expense of pleasure and emotional and relational wellbeing [17]. A retrospective questionnaire and therapy record study investigating a CBT approach found that although 81 % had achieved penetrative sex, only 6 % of participants could have pain-free intercourse at follow-up; despite this, two-thirds reported high satisfaction of individual treatment goals [18]. This suggests performance and other goals, such as pregnancy, are also important to women seeking help. Uncontrolled studies investigating the use of physical therapy for vaginismus and dyspareunia have had promising results [19–21], and an internet survey suggested women found physiotherapy the most helpful intervention [22] however the sample was small and access to physical therapy is not currently widely available.

A mixed-methods questionnaire survey [23, 24] revealed a broad range of psychosocial consequences of vaginismus [23] exacerbated by difficulties seeking help and general dissatisfaction with treatment approaches [24]. A quantitative internet survey suggested that a broad range of interventions such as physiotherapy, educational pelvic examinations, and talking about the meaning of penetration problems may be helpful, although this was a quantitative study and did not allow women to expand on what aspects of interventions women found helpful or unhelpful [22]. Although there is growing recognition of the importance of qualitative research for improving gynaecological care [25], women diagnosed with vaginismus remain a neglected population. Despite the controversies and disappointing outcomes from the few existing RCTs, women’s voices have been mostly absent from debates. This study set out to hear these voices and explore women’s experiences of treatment with VTs, with the intention of proposing a new set of guidelines to encourage improved treatment delivery, and hopefully, outcomes.

## Methods

Ethical approval was granted by the University of Nottingham and the East Midlands - Derby National Health Service (NHS) Research Ethics Committee. Participants from the community were recruited through two online vaginismus forums, and through posters displayed in clinic areas and in women’s toilet cubicles at the university. Participants were also recruited by a psychosexual therapist specialising in vaginismus and a consultant gynaecologist from their NHS clinics. The aim of recruiting through different approaches was to obtain a broad sample, reflecting various resources available in different areas, providing an opportunity for triangulation of sources [26].

Recruitment took place between August and December 2012. Eligible participants were: English-speaking women aged over 18, diagnosed with vaginismus, who had been offered or used VTs to treat vaginismus within the last ten years. All participants gave informed written consent. There was no upper age limit and women with co-morbid conditions were not excluded. This reflects naturalistic clinical populations.

Recruitment ceased after 13 interviews, as an acceptable level of data saturation was reached. Saturation (the point at which further data collection is unlikely to lead to new insights) has been described as an ‘elastic’ concept, and the desirable degree of saturation depends, in part, on the nature and breadth of the research question [27]. However, a study investigating saturation proposed 12 interviews to be adequate for most purposes [28].

Data were collected through audio-recorded semi-structured interviews conducted by a female researcher (KM). Women were given a choice of interview format (face-to-face, telephone or online - using Skype) and location (participant’s home, the university, or at the clinic). Women were assured confidentiality and had the option to pause, stop the interview or skip uncomfortable questions, although none found this necessary. Collecting data from these different types of interview formats was not considered a limitation (in terms of affecting quality, quantity, or type of information shared), but as a strength (in terms of allowing women to choose a format that they were most comfortable with, and to facilitate recruitment of a geographically diverse sample).

The interview schedule was developed in consultation with an experienced clinical psychologist and qualitative researcher (RdN) and an experienced psychosexual therapist (AG). Clients attending a specialist service were also consulted regarding the content of the interview schedule and study information packs (by AG). Interviews followed a basic structure starting with an overview of experiences, moving towards future-focused questions and ending with an opportunity to speak freely (see box 1). Spontaneous follow-up questions were used to explore participants’ answers.

Audio recordings were transcribed verbatim and analysed using Thematic Analysis (TA) [29] from a critical realist perspective [30]. Initial coding (by KM with advice from RdN) aimed to identify the smallest relevant units of meaning [31]. Codes and themes described mainly manifest (directly observable) but also latent content (issues underlying or implied by observable phenomena) [29, 31]. Initial codes were assigned to groups that were coded as sub-themes. These sub-themes were coalesced by KM and RdN (and disputes related to coding were resolved through discussion) into themes that captured information relevant to the aims of the study. The resultant thematic map was further refined through consultation with the other authors. From the thematic analysis, we were able to distil a *guide for good practice* (This guide can be found in Additional file 1), which was refined following feedback from the participants.

Ethical approval was granted by the University of Nottingham (Ref: 12065) and the East Midlands - Derby NHS Research Ethics Committee (Ref: 12/EM/0290 date of approval: 13.08.2012), and Nottingham University Hospitals Trust (Ref: 12CP009, date of approval: 28.08.2012). Permission to advertise the study was sought and obtained from the moderators of the two online communities where non NHS participants were recruited.

## Results

Thirteen women were interviewed, all identified as Caucasian. The women had a broad range of experiences in terms of their attempts to seek treatment, the majority were still undergoing or seeking treatment. For details see Table 1.

**Table 1:** Participant details

Age	Occupation	Nationality	Religion	Vaginismus Subtype
24	Research Assistant	American	None (raised Catholic)	Primary Partial
31	Visitor Assistant (museum)	British	Christian	Secondary Partial Situational
23	Volunteer/Artist/Missionary	American	Christian (Protestant)	Primary Partial
31	Data analyst	British	Christian	Primary Partial/Total
21	Student/Barmaid	British	Christian (Methodist)	Secondary Partial Situational
41	Administrator	British	None	Primary Total
67	Retired Natural	British	Christian (Methodist)	Secondary Total
	Health Therapist			
39	Research scientist	British	None	Primary Partial
31	Freelance	British	Not practising (Greek Orthodox)	Primary Total
	Designer			
51	Not working/carer	British	Not practising (Catholic)	Primary Partial
20	Student	British	None	Secondary Total/Partial/Situational
26	Unemployed(IT)	British	Agnostic	Primary
23	Waitress	American	Roman Catholic	Primary Total

Due to the broad and contested nature of the vaginismus diagnosis, participants were asked to report their symptoms. All reported pain in addition to penetration difficulties. Presentation was categorised using pre-existing criteria [11] according to the degree of penetration difficulty (penetration totally impossible, or some partial penetration possible) and whether difficulties were lifelong (primary) or secondary (occurring after a period of problem-free intercourse), in addition to whether or not difficulties occurred in all penetration attempts or differed according to situation (e.g. the woman could allow examination but not intercourse). However this was complicated as women had experienced improvements and deterioration in symptoms over time and some could allow varying degrees of penetration depending on the context. See Table 2.

**Table 2:** Participants experiences

Participant number	Summary of experience
1	First contact with GP, self-directed use of VTs followed by short term physiotherapy using VTs - not yet recovered.
2	First contact with relate (“Marriage Guidance) reassurance but no support given. Several years later saw GP, referred to NHS clinic, not helpful. 20 years later successful self-treatment.
3	First contact with GP, referred to relate, not helfpful. Self-directed use of VTs. Waiting for referral to gynecology - not yet recovered.
4	Vaginismus caused by rape. First contact with GP, referral for counselling - didn’t go because of offensive referrral letter. Went to relate, sensate focus and VTs not helpful. Referred to counselling, not helpful. Referral to psychosexual nurse, not helpful. Awaiting referral to physiotherapy. Not yet recovered.
5	First contact with GP series of referrals to gynecology, problem not recognised. GP referral to specialists clinic - finding treatment helpful, but not yet recovered.
6	First contact with GP, referred for psychosexual therapy, but husband could not attend so therapy terminated by therapist. GP suggested Relate. but this not taken up. Used self-hynotherapy recording. Now able to have sex, but not able have internal examinations.
7	First contact GP (problem initially not recognized), self referral to relate for counselling and VTs - relationship improved, but vaginismus not resolved. Referral to specialist clinic - just started therapy, hopeful of a resolution.
8	First contact with relate counselling and VTs - successful treatment.
9	First contact with PCP, who refused PCP refused exam/ diagnosis until after marriage. Referral to psychologist.- not helpful. Did own research and used VTs in vaginismus.com programme. Referral to specialist physiotherapist. Slow progress, not yet recovered.
10	Problems caused by ovarian cyst. Repeated referrals to gynecology before vaginismus diagnosed, then referred to specialist clinic. Experiencing progress, but not yet recovered.
11	First contact PCP, problem not recognized. Referred to gynecologist advised to purchase VTs. Self directed treatment with VTs. Unable to take up physiotherapy referral due to cost. Some progress, but not yet recovered.
12	First contact GP, referral to gynecology, referral to specialist clinic - much improved able to have intercourse but still in treatment.
13	First contatc GP, referred to gynecology given diagnosis and VTs, one appointment with nurse. No progress. Requested further support, advised to persist, unable to persist due to pain. Given up on treatment. Not recovered.

As this was convenience sample of self-selecting participants, details of non-participation are not available. However, nine women who expressed interest in participating were not interviewed due to not having a diagnosis (n = 2), not having used VTs (n = 1), no further contact with the researcher (n = 5), and one cancelled the interview.

**Fig. 1:**
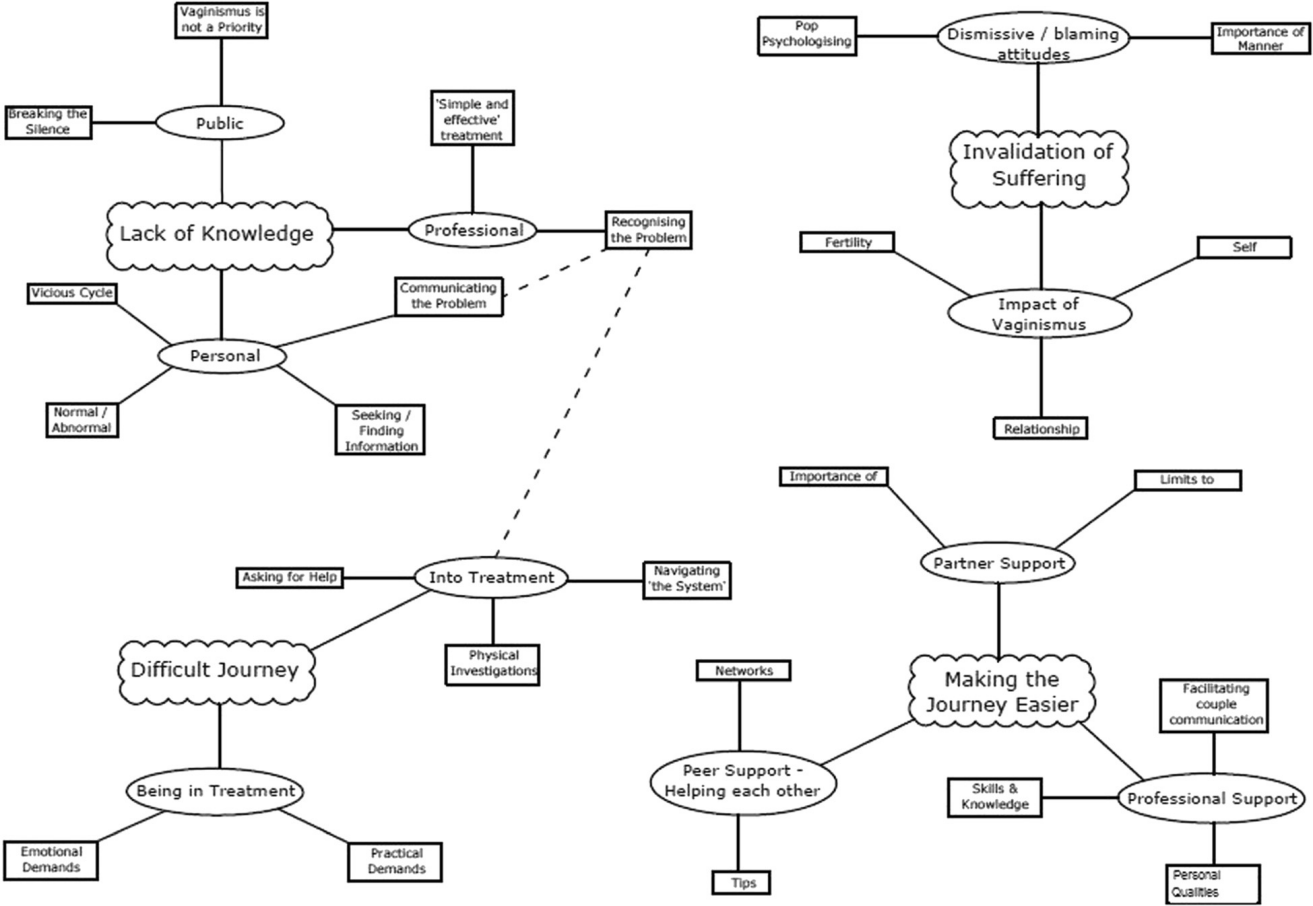
Thematic Map, showing how the subthemes covered in this paper relate to the wider research project

The final thematic map was arranged into four superordinate themes, each comprising several themes and subthemes, see Fig. 1.

Due to space constraints, we will concentrate only on two themes to provide a more detailed description and discussion of these themes.

### Difficult Journey

**Fig. 2:**
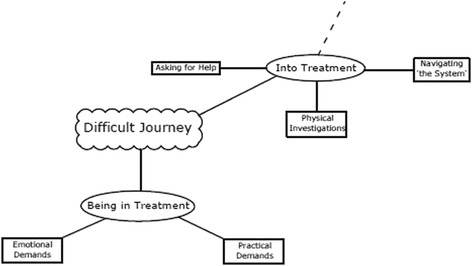
Thematic Map: Difficult Journey

Was chosen to represent the often circuitous and arduous route ‘into treatment’ and the considerable practical and emotional demands of ‘being in treatment’. See Fig. 2.

#### Into treatment

##### Asking for help

For the majority^1^ of women, their first attempt at help-seeking involved their General Practitioner (GP) or Primary Care Physician. Women described having to work up the courage to ask for help, overcoming feelings of embarrassment and abnormality, and doubts about the legitimacy of their problem. Many practitioners did not diagnose vaginismus as the problem during initial consultations and gave women advice about lubricant and foreplay and general reassurance. Unfortunately, if women felt they were not taken seriously during initial attempts to seek help, it took years before some of them felt able to seek help again.

*I was too embarrassed to… Or not… I wasn’t 100 % confident if I was actually… what I was experiencing was just normal, or something unusual that I needed help for, so I did it at the same time as doing something else. So I didn’t go in to speak to her about it, I went to her when I needed to get my pills redone, and asked her then, ‘Oh, and by the way’. P7:3*^2^

*When you are at home you are like 'Right, I am going to tell him, I am going to tell him' and when you go there you don't. P5:18*

*It didn't resolve - clearly - because these things don't necessarily, and so later I went to my GP and that would have been probably three or four years later. P2:1*

##### Physical investigations

Physical examinations presented difficulties for the majority of participants who described them as ‘*painful*’ (P6:3), ‘*humiliating*’ (P5:6), and even ‘*traumatic*’ (P12:2). Two women had requested to have partners accompany them during physical examinations, but were told this was not allowed. For some women physical examinations were not possible due to pain and muscle tension. Some practitioners had worked around this, doing examinations under general anaesthetic, which the women appreciated. One woman had never had a successful physical examination, and had been diagnosed with vaginismus because of this. She had requested an examination under anaesthetic to rule out other problems, but this was refused.

*You lie down in a specific position that makes you feel quite vulnerable, and there is a doctor standing at the opposite end of you and she is trying to stick her finger in you [both laugh]. And then it doesn't go in and she is saying 'Relax. Relax. Relax'. P3:17*

*They tried to give me a smear, the nurse, but I was in too much pain. So then I went to see a female doctor, who just looked at me… she hardly touched me and I just screamed. P6:3*

*It was all a bit difficult, but then after that, when I went back, I went back for the examination under anaesthetic and the nurses there were super, and afterwards he explained to me what he’d found. P13:2*

Three women had been repeatedly tested for sexually transmitted diseases, which they found disrespectful. All stated that testing caused them pain, which made them reluctant to cooperate when they saw the tests as unnecessary.

*It was like they were calling me a liar I suppose… I would say to every single one 'I have already been tested, you can test me whenever you want…' but no it was get on the couch and do all the plastic and crap and send it off. P5:14*

Several women did, however, have positive experiences of physical examinations and were able to describe professional approaches that made this possible.

*Just being really clear about what’s going on during the exam and showing me yes ‘we are using the paediatric size speculum because I know that’s an issue’ ha ha, and, yes just I mean I guess just mainly the personality that thing, but just being able to talk about any of it, like ‘I’m putting it in, now I’m going to open it up, you know I’m doing the PAP smear bit now, I am going to tap here’… It just sort of made it a whole lot easier to deal with than just kind of like routine ‘okay legs open, ready?’ P11:7*

##### Navigating the System

Many women had seen several different professionals to find help. For some, this led to a series of disappointing encounters or failed treatments. Others had been referred directly or eventually to a specialist who could help. This depended on the referring practitioner taking time to find, or already knowing of, specialist local resources, but also on the availability of local resources to refer to.

*My doctor was just so fed up, because he'd referred me to a few people and he said, he sort of said ‘why do I have to keep writing all these letters?’ P4:6*

*You go into a system, then they kind of put you away somewhere… I think the fact that he looked and found where he could choose to put me, that was nice. I’m grateful I’m there, definitely that he took the time. P5:12*

*I mean like now it is as if they don't want to help, they want someone else to help, which is a bit annoying because when our local hospital doesn't have a therapist anymore, they are having to apply for funding for me to see someone in the next town. So I have got to wait for all that. P6:5*

Some women had been referred to non-statutory agencies (or had sought these services out themselves) and they had mixed experiences. For some, cost prevented them from using services. For participants from the USA navigating insurance systems was also difficult.

*Because we were paying for the therapy, it was private, we just… I thought that I had learned enough and that I had accepted the problems that I had. P8:5*

*[the doctor] referred me to a … therapist here which I can’t afford to go to, I mean there is no way, but I called them and I told them about my insurance and they told me it was probably going to be between $150 to $200 per visit. P11:10*

#### Being in Treatment

##### Practical demands of therapy

Women discussed the practicalities of obtaining suitable VTs. While some had been given VTs by a practitioner, others had been encouraged to buy their own from the internet or a sex shop. Some women described finding the smallest trainer too big or being unable to transition from one trainer to the next due to the jump in size. Some women sought alternatives such as vibrators or dildos, or used smaller items such as cotton buds, fingers or tampon applicators. Finding privacy and time to use VTs (or alternatives) was also a problem.

*[the Doctor] went and looked online, you know, Vaginmus.com, and stuff I mentioned to her, and she looked at it, and she said, ‘Well this sounds like a great idea, why don’t you try ordering some for yourself?’ And I did, but of course she wasn’t able to instruct me in how to use them. P1:7/8*

*When I first started using them [VTs] I was living with my parents, so it was a little awkward at the time, I wasn’t really sure how to do that without them, finding out. And my mother never liked to knock, so that was always a problem. P1:9*

*Sometimes it’s a toss-up between doing some work or doing my trainers and I choose work [laughs], over trainers. P10:12*

Lack of knowledge of how to use VTs also caused problems. Women feared making things worse or wasting their time by not using them properly, but found it difficult to obtain technical advice about what to do. Some had been advised to start with non-penetrative exercises, however others had been advised to continue *despite* experiencing intense pain and failure when trying to insert the smallest VT.

*Because I tried once, and never, never again, because I had no instruction or I didn't know what to do. So they [VTs] have just stayed in the bag, stayed in the drawer and I wouldn't do it again, but… until I saw the woman I am seeing now and she asked me about them. I said I have got some, and it turns out I have got the wrong ones. P5:7*

*Every time I tried it [VT] was almost as if I was torturing myself. P13:18*

*I went back once and I said ‘I am still having the same difficulty’ and the advice I was given ‘well just carry on trying and eventually you will find that it will help you’ and that was it [laughs]. P13:8*

##### Emotional Demands of Therapy

Several women were disappointed to learn that there was no ‘magic pill’ (P2:5) or simple cure for vaginsimus.

*It takes a lot of patience and you know when you read some testimonies on the site of some women saying that they completed the entire programme and within 3 months [they were better], that’s the hope that you have when you first get it. P9:8*

*So I thought oh wow! I am going to go and see a therapist who knows exactly what they are talking about, and she is going to be able to cure us in two weeks. But obviously it wasn't like that. P8:7*

Some women were upset or frightened by the VTs, while others were angry about ‘having’ to use them and not being able to just have sex ‘naturally’. Several described how it took time to get used to them, while others did not get used to them at all.

*It’s kind of an event when I do it erm, it’s less of an event than it used to be… I was kind of terrified for a while. P11:6*

Having to use VTs regularly meant that women were continually forced to confront the problem, and what it meant to them.

*And then we would go back and be all embarrassed and report that everything was fine, but I think they believed that where everything was fine and we were doing everything we should. But it wasn't fine; it was only ever painful and horrible and awkward and uncomfortable, and embarrassing. P2:4*

*Instead of like having to do this every day and trying to because it’s not really something I want to think about like. It’s not a very womanly thing to be broken, [laughs], but I don’t know I just try and push it to the back of my mind. P10:4*

*I probably had one of the worst times of my life was going through this therapy and this sort of trauma. P3:17/18*

Progress, if any, was slow, and women needed a sense of progress to stay motivated to do exercises. This was compounded by feeling unsure about what to do or what to expect.

*Needing a sense of progress, I guess just to make sure you know I had some questions at the beginning like am I doing this right, what’s supposed to be happening, am I supposed to be feeling, I don’t know, am I supposed do it so that I don’t feel any pain at all or you know, should I try to almost like trick the muscle a little bit and slip it in before anything happens or should I go really, really slowly and, I guess yes, that was a concern for a while that I was doing it wrong and I was wasting my time. P11:7*

### Making the Journey easier

**Fig. 3:**
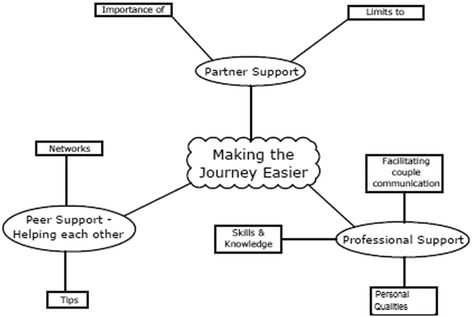
Thematic Map: Making the Journey Easier

The women’s experiences left them with a wealth of knowledge. They felt that by telling others what facilitated their treatment journey, women could take some control of their recovery. They also hoped by taking part in this research they could perhaps improve treatment for others. See Fig. 3.

#### Partner support

##### Importance of partner support

Women described how important it was to be in a good relationship and to have a supportive partner.

*My Partner has been amazing, because he, he bugs me about it; he is like [jovially] ‘Oi! I want to have sex! Use your trainers!’ So that’s very helpful. P10:12*

*If you have got this condition, you need somebody who is behind you every step of the way because it’s… it’s a traumatic experience, that affects you and if they don’t understand, then its not going to help you at all…So as I say you know, like any women who have got it, if they’ve got a supportive partner it makes all the difference I think, definitely. P12:15*

##### Limits to partner support

However, it could be difficult for partners to understand vaginismus and several women described tolerating painful sex (unknowingly making the problem worse) because they felt that it would be ‘unfair’ to deny their partner sex.

*Then when you’re trying to sort out other problems, it gives… it sort of says, ‘well why should he have to sort out the problems that we’ve got if… Why should he make changes if I’m just not making an effort in sex?’, because that’s how it felt to him. P7:21*

*I was trying to cope with the pain to be fair to my husband and I couldn’t, I couldn’t fulfil what I felt …not just that I felt I should, I mean we’d had a really good sex life until then, and I missed it. P13:10*

Some women described their partners becoming fearful of hurting and upsetting them through past experiences, or that a sense of fear and awkwardness made it difficult for them to discuss the problem as a couple.

*He also seems kind of afraid. Like I’ve asked him a couple of times if he would use the dilators with me, and he just… he says, you know, ‘I don’t want to hurt you. I don’t want to do something that’ll make you feel bad about it’, since he would be the one there using them. I’ve tried to coax him and do it a couple of times, but he is… he… I guess he is worried. P1:12*

#### Professional support

##### Importance of practitioners’ personal qualities and the therapeutic relationship

Many women described professionals they found particularly helpful. Practitioners who listened non-judgementally and empathically were particularly valued. Women were grateful to those who believed them and did not send them away.

*She [doctor] is genuinely such a nice person, and she is not up tight, and she will just sit back and talk to you like you are a human I suppose. P5:15*

*Their [clinicians’] approach was really genuine, and they didn’t make me feel as if I was making a fuss, they didn’t make me feel, which I have had from other people from time to time that it was a bit of a fuss for somebody for my age to be worrying about things like that. P13:15*

##### Value of specialist skills and knowledge

Women valued specialist expertise, and practitioners who offered specific advice about exercises, training in relaxation techniques, and who were knowledgeable about effective treatments.

*They have dealt with this before, they’ve dealt with plenty of people with the same problem, and they have techniques which they have… which have proven to work in the past. P7:18*

Women acknowledged that practitioners ‘can’t know everything’ and appreciated those who took the time to do some research.

*She learned I think she… you know, she heard from me, and then went and did research on her own, which was great, like I really, really appreciated that, that she did that. P1:16*

##### Facilitating couple communication about vaginismus

Some participants described difficulties approaching the problem within their relationship and welcomed flexible support from practitioners who helped enhance their communication without blaming their relationship.

*I will say sometimes [to partner] 'Do you want to come this week, we could do with talking?' Or I will say 'I don't mind going on my own', kind of means that I need to talk to her [clinician] on my own, so. No, at first I was afraid to go on my own, and then I kind of sucked it up and thought I will try it and see what it was like, and it turned out actually it is really helpful being able to go on my own sometimes, especially when I want to talk about trainers and stuff it is a lot easier [laughs]. It is nice having the choice. P5:16/17*

*I think to take it off of me as well. So obviously we were doing it… my boyfriend and I were doing it together. I think it was to bond both of us. So if she concentrated just on me, which is obviously a large part of the therapy perhaps he would feel excluded as well. P8:10/11*

Attending with partners was not always possible, and one woman felt ‘devastated’ (P6:3) when a therapist refused to see her because her partner could not come.

#### Peer support/helping each other

##### Networks

Several women found contacting others diagnosed with vaginismus helpful, for example through online forums. Women described a need to meet people who understood; and one woman stated she would like face-to-face support groups to be developed. Some women also appreciated the opportunity to *give* support to others.

*I guess the like being able to use the Internet and research it and look on Forums and stuff and find other people that are having the same problems and yes, do find like using the trainers like tedious and whatever, its good to know that there is other people out there. P10:12*

*Just people that understand exactly what you are going through that don't judge you for what you are saying, and don't think you are a freak because they understand. P3:15*

##### Sharing tips

In addition to the emotional support, women were able to gain and share advice about what had helped them or other people.

*You know, I think I got more information about breathing and different positions and like angling it properly… I really got it off the internet to be honest. P11:8*

*Well I told a friend, who knew of a friend who had it, who had used the self hypnosis and it is like a… I downloaded it as an MP3. P6:16*

## Discussion

The women in this study revealed vaginal penetration difficulties can be isolating, and professionals do not always respond appropriately. These findings resonate with findings from other studies also [20, 21]. Our study further explored perceptions of treatment barriers, including difficulties asking for help, problems with physical investigations, and difficulties navigating complex, and sometimes unsympathetic, medical systems. Not knowing whether pain was ‘normal’ hindered women’s help-seeking, and professionals’ recognition of vaginismus, or indeed other causes of vaginal penetration difficulties which women in this sample may have been experiencing. In contrast to the easy solution women often hope for, treatment requires time and commitment which can be difficult to maintain, practically and emotionally, especially when progress is slow or non-existent. Women had tried using alternatives such as their own or partner’s fingers, or vibrators, to overcome feelings of the dilators being ‘unnatural’ or ‘clinical’, however this did not alter difficulties regarding size and the time and commitment necessary for progress. Lack of information about how to use VTs or alternatives effectively was a further practical difficulty. It was particularly concerning that women had persisted with, or had been advised to persist with, using VTs in ways that caused pain. This suggests not just absence of information, but the presence of potentially harmful misinformation among practitioners. Women’s experiences of physical examination mirrors the strategies reported by professionals who have experience of women who are difficult to examine, although guidelines for more therapeutic examination do exist [32].

The treatment was considered emotionally demanding as it forced women to confront the realities of their penetration difficulties and how these difficulties made them feel about themselves. For women whose problems were related to abusive sexual experiences, this could be additionally distressing. Discussing these aspects of the treatment with professionals helped women come to terms with needing to use VTs. However, progress was also important for maintaining motivation.

Women appreciated knowledgeable practitioners who listened non-judgementally, understood their needs, and offered support and effective treatment. Specialist skills and knowledge were highly valued, as was the ability to enhance couple communication and facilitate couple cooperation in overcoming vaginismus. Partner support was important. However this was limited due to partners having also experienced negative conditioning, and also lacking knowledge about the condition and its treatment. The internet provided a useful resource for some women who shared information and provided mutual support to each other.

This study goes beyond previous research by elucidating the experience of treatment with VTs. These findings must, however, been seen in the context of the limitations of this study. Recruiting women with the diagnosis of vaginismus was intended to mirror research conducted into the experiences of women with other sexual pain disorder diagnoses [33, 34]. Recruitment took place before publication of the DSM5, and so the new umbrella diagnosis of genito-pelvic pain/penetration disorder (GPPPD), which arguably better reflects the diverse symptoms of the sample was not available. Diagnoses were made by clinicians independent of the research team, and in some cases women with symptoms suggesting vaginal atrophy and provoked vulvodynia were offered treatment for vaginismus that in isolation could not be expected to help them. Relying on diagnoses from independent professionals is a significant limitation of this study. However, while the finding that the diagnosis is being applied so broadly is unsurprising given the debate in the literature, the finding that women are potentially being offered inappropriate treatment due to lack of professional knowledge is extremely important and very worrying. This underlines the need for the development and effective dissemination of professional guidelines for the assessment and treatment of women experiencing penetration difficulties and for more rigorous service evaluation to ensure quality and appropriateness of current treatment. Further research recruiting participants from the entire spectrum of GPPPD could facilitate further clarification of what works for whom.

Using diverse recruitment sites was intended to mitigate against reliance on convenience sampling of self-selected individuals, but details of non-responders are not available for comparison. All participants identified ethnically as White, and the majority were from Christian backgrounds. Further research is needed to ascertain whether similar themes are applicable to women from other socio-cultural groups. The sample cannot be assumed to be representative of the wider population of women with diagnosed vaginismus, however the presence of certain themes across the different recruitment and geographical contexts suggests that these themes may be relevant to other women seeking and receiving treatment.

As with all research, data were interpreted through the ‘lens’ of the researchers [35]. Efforts were made to make the analytic process as transparent and standardised as possible, and quality control was carried out by two authors, to ensure interpretations did not go beyond what could reasonably be said to lie within the data. That said, meanings cannot be measured, they can only be understood, and understanding will always be influenced by an individual’s history and world view [36].

Earlier detection may reduce treatment time and reduce the financial cost of repeated unhelpful appointments, as well as reducing the psychosocial costs of the disorder.

However embarrassment and awkwardness may be shared by primary care practitioners who feel insufficiently skilled [37]. Unclear advice about how to use VTs effectively may relate lack of clarity in the existing literature [9]. Specialist referrals may be necessary for women whose vaginismus is related to emotional trauma [10]. Progress aided motivation, suggesting self-efficacy models may be informative in enhancing treatment outcomes [38]. Learned helplessness models may also explain how lack of progress can lead to giving-up on treatment or using VTs inconsistently [39]. A focus on treating women individually may explain why the levels of success described by Masters and Johnson [14] have not been replicated in recent RCTs [15]. Formal peer support has been used successfully with other chronic pain populations and may be a cost-effective way of expanding service provision [40]. However less formal structures might also be beneficial.

## Conclusions

Accessing effective treatment for vaginal penetration difficulties is difficult. The practical and emotional demands of using vaginal trainers may be underestimated by professionals, resulting in inadequate provision of support and information in practice. At times vaginal trainers may be prescribed to women who are unlikely to benefit from this treatment in isolation. Core communication skills like non-judgemental listening are important for supporting women through treatment. However professionals also need greater specialist knowledge, which in turn requires more detailed research.

## Additional file

Additional file 1
**Guide for good practice approved by particpants.**

## Abbreviations

VTs: Vaginal trainers
TA: Thematic analysis

## Footnotes

1 We use both numeric (1, 2, 3, etc.) and quantity terms (some, majority, etc.) only as narrative descriptors, but not for the purpose of quantification.

2 The participant number is indicated by a number following P (P1-13); the number following the colon indicates the transcript page number.
