# Supplementary material for: Women’s experiences of using vaginal trainers (dilators) to treat vaginal penetration difficulties diagnosed as vaginismus: a qualitative interview study
Source: BMC Womens Health. 2015 Jun 20;15:49. doi: 10.1186/s12905-015-0201-6 (PMC4475318; doi:10.1186/s12905-015-0201-6)
Supplement: Additional file 1 — Guide for good practice approved by particpants. [file 12905_2015_201_MOESM1_ESM.doc]

# Women’s experiences of using vaginal trainers (dilators) to treat vaginismus: A qualitative interview study

Kat Macey^1^, Roshan das Nair^1,2^, Angela Gregory^3^ and David Nunns^4^

^1^ Trent Doctorate in Clinical Psychology, University Of Nottingham

^2^ Dept. of Clinical Psychology & Neuropsychology, Nottingham University Hospitals NHS Trust

^3^ Chandos Clinic, Nottingham University Hospitals NHS Trust

^4^ Division of Obstetrics & Gynaecology, Nottingham University Hospitals NHS Trust

Corresponding Author: Kat Macey [lwxkjma@nottingham.ac.uk](mailto:lwxkjma@nottingham.ac.uk)

Trent Doctorate in Clinical Psychology, University of Nottingham, Jubilee Campus, Triumph Road, NG8 1BB

# Draft Guidelines for improving vaginal trainer treatment for vaginismus

These guidelines were developed through semi-structured interviews with women who had used vaginal trainers (dilators) to treat vaginismus. They are intended to provoke further debate and discussion and ultimately to improve treatment experience for affected women.

This document is intended to be useful for the range of professionals likely to come into contact with women with vaginismus at some point in their career. The women who took part in this research identified a need for societal level change, which will be beyond the scope of many professionals; however guidelines concerning this have been included to reflect this need. Some of the guidelines require specialist expertise, and therefore liaison with colleagues from other disciplines is advised.

This document has been circulated among the women who took part in this study, and adapted in response to their comments.

## Guidelines for facilitating earlier recognition and access to treatment

### a) Use Continuing Professional Development (CPD) events and other opportunities to educate colleagues and trainees/ take advantage of opportunities to learn more.

Professional education is needed to help professionals recognise the problem and the diversity of vaginismus presentations, including difficulties women may have in describing the symptoms. This would reduce the number of appointments needed to obtain a diagnosis, help women to get treatment earlier (reducing psychosocial and reproductive consequences) and reduce the length of time women are exposed to sexual experiences that exacerbate the problem and make it more difficult to treat.

### b) Use public health events and other opportunities to educate members of the public

Public education is needed to help women to recognise the symptoms and facilitate help-seeking, by reducing fear of the symptoms and embarrassment about seeking help.

### c) Use posters and leaflets to ‘give permission’ for women to raise sexual concerns – ask about sex.

Professionals need to make it easier for women to talk to them about vaginismus, for example by having posters or leaflets in doctor’s surgeries (usually the first point of contact). Professionals should be aware that women can find it hard to talk about sexual difficulties and may put off help-seeking, lose courage and not mention the problem, or mention the problem at the end of an appointment about something else. This does not mean it is unimportant to them.

### d) Attitude and manner are important

Women seeking help may be feeling very vulnerable and may present as highly distressed. It is important that you listen to and respect them and that your body language also shows this. Do not imply that you think the woman is lying, or it is ‘all in her head’ or that women should just accept painful sex. Accept that the problem is real and important.

## Physical Examinations

### a) Rule out or address other problems

Physical examinations are necessary at some point to rule out or identify other conditions that may be causing the problem (but see below).

### b) Do not pressure women to have an exam

Trust is very important, if a woman has had negative penetration experiences previously it may take several consultations before the woman can relax enough for physical examination to be possible. Examinations can be traumatic if they are done unsympathetically, under pressure, or when the woman is too anxious, in too much pain or cannot relax her muscles sufficiently.

c) Examination under anaesthetic may be necessary

The risk of harm to the woman from undergoing anaesthetic may be less than the harm of leaving physical conditions untreated. Traumatic exam experiences, repeated unsuccessful exams and long delays to treatment are believed to exacerbate symptoms and make them harder to treat. Women can find the process of failed exams ‘humiliating’, this can damage relationships with professionals.

### d) Go slowly, and describe and ask permission before completing each step of the exam.

If an exam is appropriate, it is important that the woman knows what you are going to do at each point, and agrees that this is okay. Tell her you will pause or stop completely at any point if she wants you to. Go slowly, use a small speculum and explain each step and ask permission before you do it. Do not expect the woman to take in a lot of information at once if she is anxious. Give an overview, but you will need to repeat things to make sure she is giving informed consent for each step.

### e) ‘Vaginismus’ covers a diverse set of symptoms.

Some women can undergo examinations without problems and their symptoms may only be present in sexual situations. This is called ‘situational vaginismus’.

## Ensure women receive effective treatment

### a) Address physical concerns

Other physical problems that make penetration painful such as vulvodynia, ovarian cysts and post-menopausal syndromes must be addressed as women cannot learn that penetration is not painful, when they are continually confronted with pain.

### b) Refer to specialist services if possible

Where possible, referral to specialist services with experience of treating vaginismus is preferred to generic sexual health or mental health services, which have varying levels of experience. NHS services are preferred to private sex therapy in the UK, due to potentially prohibitive costs and considerations of feeling more abnormal and less cared for when no statutory services are available. Health insurance may be barrier to treatment for women in some countries.

### c) Support women to say ‘No’ to painful sex

Women should be advised to stop painful activities (but continue with non-painful sexual activities) due to the risk of making the condition worse. Leaflets may be useful for explaining this to partners, but joint sessions may be needed. Some women may be able to ‘self-treat’ if given appropriate information at this stage.

### d) Support women to choose an achievable starting point

Women need support to choose an appropriate starting point. Some women may need to begin with non-penetrative exercises or sensate focus exercises with a partner. Vaginal trainers come in a restricted number of sizes and the smallest may be too big for some women, alternatives such as well lubricated cotton buds or tampon applicators may be useful. A sense of progress is important for motivating persistent and consistent use of trainers, it is therefore better to start with steps women know they can accomplish easily.

### e) Help women obtain suitable vaginal trainers (dilators)

Vaginal trainers are available on prescription in the UK. There are a range of trainer sets available online, but some are expensive and women may need support to choose the most appropriate kind. Some women do not have access to the internet and may need to use a local sex shop. Trainers with a graduated tip are preferable to flatter ended trainers. Vibrators and dildos of appropriate sizes may be preferable for some women. Lubricant is necessary, high quality lubricants are preferable.

### f) Help women learn specific relaxation techniques

Specific relaxation exercises, such as breathing exercises and audio recorded guided self-hypnosis can be more helpful than general statements that it is important to relax.

### g) Give detailed and specific instructions

Give a clear rationale for using trainers, e.g. to ‘get used to’ the feeling of penetration and to ‘teach the body that penetration is not painful’. Detailed instructions about using trainers such as, avoiding pain, angles and positions, inserting on the exhalation, etc. are helpful. More research and better communication among professionals and successfully treated women may lead to more effective programmes.

### f) Assess and treat pelvic floor muscle dysfunction

Physiotherapists can help women to identify, exercise and gain greater control over affected muscles.

## Support women with the emotional impact of treatment

### a) Prepare women with an accurate picture of the task ahead

Prepare women concerning details of the likely length of treatment, the need for persistent and regular practice, the possibility of finding using trainers emotionally difficult (because it means confronting the problem on a daily basis), and the practical inconvenience of working with trainers. For some women progress is slow.

### b) Give women time and support to adjust to treatment

Women may find trainers clinical, unnatural and frightening. It is important to allow women time to adjust and get used to the idea and not to pressure women to use them until they are ready. Using their fingers may be preferable for some women, but others find them uncomfortable. Help to overcome this in small steps such as handling the trainers during an appointment may be helpful.

### c) Provide ongoing support (don’t just discharge)

Follow-up appointments are important to help women remain motivated and to help problem-solve any difficulties that arise. A flexible, non-judgemental and responsive approach is important for building an effective therapeutic relationship. It is important that the woman can trust you and tell you about any problems.

### d) Signpost to other resources

Vaginismus can be very isolating, sign-posting to online forums or local support groups may help reduce this and make accessing information easier. Not everyone has access to the internet.

## Involve Partners flexibly

### a) Support partners to move beyond past negative experiences

Partners may fear hurting and upsetting the woman due to negative experiences and may have their own sexual difficulties. The couple may need support to communicate about the problem and to face it together, so that learning (that penetration is safe and enjoyable) can be generalised to sexual contexts.

### b) Allow the couple to choose what support they need

Give couples some control over whether to attend appointments together or separately, there are some things it may be easier for them to address individually (e.g. past sexual experiences/ techniques for using vaginal trainers) and some things they may need to address together.

## Provide fertility assistance

Treatments for vaginismus are not perfect, and can take a long time to be effective. It may not be reasonable for a woman to wait until the vaginismus is under control before starting a family. Reproductive assistance may be necessary. Childbirth may be difficult if the woman cannot allow vaginal examinations but effective pain relief may make this easier and Caesarean section has been employed successfully.

## Promote future research

More research is needed to optimise treatment. Researchers should clarify the specific techniques being used by women in their samples.

More research is needed to provide women with realistic expectations about the likely length and outcome of treatment.

More research is needed to answer the following questions:

- Is pain during early intercourse common enough to be considered ‘normal’?
- Is pain inevitable for some women, or is this dependent on technique and potentially avoidable with better sex education?
- If pain is ‘normal’ and ‘inevitable’ how long should women and professionals wait before considering it a problem?
